# Understanding the Links among Maternal Diet, Myelination, and Depression: Preclinical and Clinical Overview

**DOI:** 10.3390/cells11030540

**Published:** 2022-02-04

**Authors:** Irena Smaga

**Affiliations:** Department of Drug Addiction Pharmacology, Maj Institute of Pharmacology Polish Academy of Sciences, Smętna 12, 31-343 Kraków, Poland; smaga@if-pan.krakow.pl; Tel.: +48-12-662-3268; Fax: +48-12-637-4500

**Keywords:** depression, maternal diet, myelination, oligodendrocyte, oligodendrocyte maturation

## Abstract

Depression is one of the most common mental disorders in the general population, and multiple mechanisms are involved in the etiology of this disease, including myelination. According to the Developmental Origins of Health and Disease (DOHaD) hypothesis, maternal diet affects the lifetime of the individual during adulthood and may contribute to the development of neuropsychiatric disorders. Additionally, the intensive processes of myelination contribute to the development of the central nervous system in the perinatal period, while any alterations during this crucial process providing the physiological functioning of neurons may lead to neuropsychiatric disorders in the next generation. The present review summarizes the current knowledge on the role of the myelin-related changes in depression, as well as the crosstalk among maternal malnutrition, myelination, and depression in preclinical and clinical settings.

## 1. Introduction

Depression is one of the most common mental disorders in the general population and affects all aspects of human life; it is characterized by sadness, guilt, loneliness, low self-worth, disturbed sleep or appetite, and loss of pleasure or concentration. Multiple potential mechanisms are involved in the etiology of depression, indicating the large heterogeneity of this disease [1]. It is now known that the etiology of this mental disorder is much more complex and related to several agents (i.e., reduced brain monoamine levels, stress, infections, genes, inflammation, etc.) [1,2], as well as disturbance within myelination during the prenatal period.

Maternal nutrition, stress, physical activity, substance use disorder, and food preferences seem to be crucial during the fetal period. The Developmental Origins of Health and Disease (DOHaD) hypothesis posits that the in utero and early postnatal experience of the maternal diet affects the lifetime of the individual during adulthood and may contribute to the development of neuropsychiatric disorders [3]. In the perinatal period, the intensive processes of myelination contribute to the development of the central nervous system (CNS) [4], while any alterations during this crucial process providing the physiological functioning of neurons may lead to neurological disorders, including depression [5].

Over the past decade, several studies have suggested that diet could play an important role in the treatment and prevention of depression, as well as the potential effect of maternal nutritional imbalance during gestation on the risk of depressive-like behavior in offspring [5,6,7,8,9,10,11]. The neonatal nervous tissue is extremely sensitive to alterations in local homeostasis, which may be interrupted by several agents, including the maternal diet during gestation and lactation. In the perinatal period, due to intensive processes of neurogenesis and gliogenesis, a huge number of neural progenitors arise, which contribute to the development of the CNS. The newly born neuroblasts give rise to motoneurons, sensory neurons, which are specialized neurons responsible for behavioral and cognitive functions, or interneurons responsible for the interactions between those cells [12,13]. Any alterations during the crucial processing and transmitting of signals within the nervous system providing the physiological functioning of neurons may lead to neurological disorders, including depression [5].

The present review summarizes the current knowledge on the role of the myelin-related changes in depression, as well as the crosslink among maternal malnutrition, myelination, and depression, and it discusses new directions in studies of depression based on a more comprehensive understanding of molecular determinants related to the myelination that participates in this brain disorder.

## 2. Myelin—Structure and Function

Myelin is a lipid-rich membrane structure produced by myelinating glial cells, the oligodendrocytes in the CNS, and Schwann cells in the peripheral nervous system. The myelin sheets are composed of (i) tightly packed spiraling layers of the membrane of mature oligodendrocytes that lack a cytoplasm, called compact myelin, and (ii) cytoplasmic noncompact regions connecting the oligodendrocyte cell body to the axonal side of the wraps and the nodes of Ranvier [14]. Unlike other biological membranes, compact myelin is made largely of lipids (70–80%) and is enriched in several proteins (20–30%) [15]. Myelin is a compact multilamellar and highly organized structure [14], which insulates nerve cell axons and facilitates signal conduction along with bigger distances as an evolutionary adaptation of the increasing body size of animals. The discontinuous structure of the myelin sheath results in saltatory conduction, whereby the action potential “jumps” from one node of Ranvier over a long-myelinated stretch of the axon. The nodes of Ranvier, as nonmyelinated axonal tracts, provide the regeneration of the neuronal action potential via voltage-sensitive sodium (Na_v_) channels required for the genesis of the action potential and subsequent repolarization using paranodal fast potassium (K^+^) channels [16]. Then, this electrical signal provokes the release of neurotransmitters [17] from both excitatory [18] and inhibitory [19] neurons, which bind to receptors on the adjacent postsynaptic cell in synapses. In addition to increasing the velocity of the action potential, myelin provides several critical functions in the nervous system, including mechanistic protection of the axon isolating the electrical signal, axonal growth, metabolism, integrity, and survival [20,21], regulation of neurotransmission [22], neuronal circuits [23], and synaptic plasticity [24].

## 3. Oligodendrocyte Differentiation, Maturation, and Myelination

Oligodendrocytes derive from highly migratory and proliferative neonatal progenitors, which appear in the developing nervous system starting from approximate midgestation. During development, oligodendrocytes undergo a multistep process of maturation to obtain the capacity for myelination. The majority of neonatal oligodendrocyte progenitors differentiate into mature oligodendrocytes, but a small number of neonatal progenitors remain and transform into adult oligodendrocyte progenitors [25,26,27,28].

**Figure 1:**
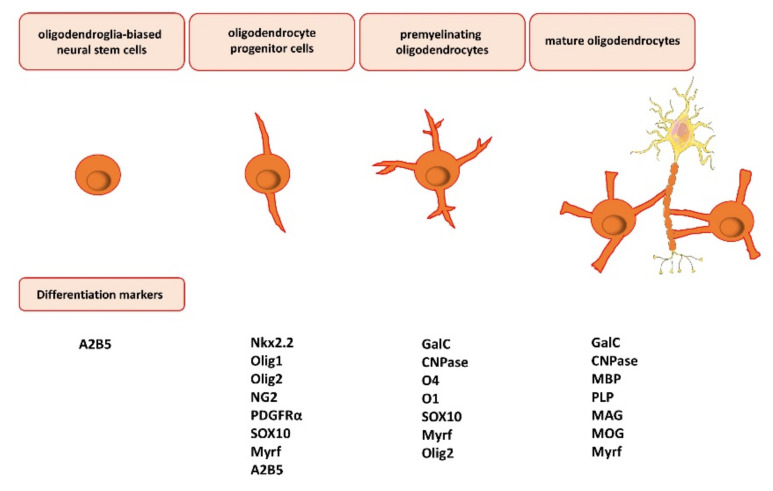
Oligodendrocyte differentiation, maturation, and myelination. The maturation of oligodendrocytes is characterized by several overlapping markers, whose expression is specific to the maturation steps. Oligodendroglia-biased neural stem cells present the A2B5 marker on the surface. Oligodendrocyte progenitor cells express NK2 homeobox 2 (Nkx2.2), oligodendrocyte transcription factor 1 and 2 (Olig1/Olig2) (nuclear or cytosolic), chondroitin sulfate proteoglycans, represented by neuron/glial antigen 2 (NG2), platelet-derived growth factor receptor α (PDGFRα), transcription factor SOX10, and myelin regulatory factor (Myrf). Next, premyelinating oligodendrocytes express the intracellular presence of 2′,3′-cyclic nucleotide-3′-phosphodiesterase (CNPase) and galactosylceramidase (GalC). Lastly, mature oligodendrocytes may be recognized by the following markers: myelin basic protein (MBP), proteolipid protein (PLP), myelin-associated glycoprotein (MAG), and myelin oligodendrocyte glycoprotein (MOG).

The maturation of oligodendrocytes is characterized by several overlapping markers, whose expression is specific to the maturation steps [29] (Figure 1). The capability for myelination is related to the activation of several genes coding for specific protein and lipid components. Most oligodendrocytes develop during embryogenesis and early postnatal life from restricted periventricular germinal regions [30], and glial fibrillary acidic protein (GFAP)-positive astrocytes (type B cells) also generate a small number of nonmyelinating oligodendrocyte precursor cells and mature myelinating oligodendrocytes [31]. Oligodendroglia-biased neural stem cells present the A2B5 marker on the surface [32], while oligodendroglial progenitors are characterized by the presence of transcription factors, i.e., the homeodomain protein NK2 homeobox 2 (Nkx2.2) [33], oligodendrocyte transcription factor 1 and 2 (Olig1/Olig2) [34], SOX10, and myelin regulatory factor (Myrf) [35]. Oligodendrocyte progenitor cells also express platelet-derived growth factor receptor α (PDGFRα) [27] and the transmembrane chondroitin sulfate proteoglycans represented by neuron/glial antigen 2 (NG2) [36], which are involved in cell migration and the reaction in pathological states. Immature oligodendrocyte progenitor cells NG2-positive (NG2^+^) with branched cell processes may remain in their undifferentiated state with a high proliferative potential in the CNS [37]. Differentiation of oligodendrocyte progenitor cells, which are the ultimate precursors of myelinating cells, is characterized by the sulfatide O4 and O1 markers (pre-oligodendrocytes) [38]. After this stage, the differentiated oligodendrocytes form premyelinating and myelinated oligodendrocytes. Maturing cells are recognized by their two most characteristic markers: the intracellular presence of 2′,3′-cyclic nucleotide-3′-phosphodiesterase (CNPase), contributing to myelin synthesis and maintenance, and galactosylceramidase (GalC), providing the hydrolysis of certain galactolipids, which are integrative myelin molecules, while the presence of O4 in these cells vanishes [39]. Mature oligodendrocytes and the formed myelin sheaths may be recognized by the following markers: myelin basic protein (MBP), proteolipid protein (PLP), myelin-associated glycoprotein (MAG), and myelin oligodendrocyte glycoprotein (MOG) [40] (Figure 1). Myelin sheaths are very variable in patterning and coverage due to the fact that oligodendrocytes may form different numbers of myelin sheaths characterized by different lengths and thicknesses [14].

In the CNS, myelination is accomplished by oligodendrocytes that are subjected to several processes including flattening, wrapping around the axons, and widening and elongating to form a myelin internode [41]. Polymerization of actin-related protein 2/3 complex (Arp2/3) is required for the first step of myelination [42], while actin depolymerization factor (ADF)/cofilin-1 is needed for the wrapping of the myelin [43]. The latter process is also endorsed by the CNPase, which prevents myelin compaction for extension of the myelin sheath [44]. Myelin thickness is regulated by the growth factors brain-derived neurotrophic factor (BDNF) and basic fibroblast growth factor (FGF2). FGF2, via the activation of the extracellular signal-related kinase (ERK) 1/2, targets the transcription factor Myrf, in addition to activating the mechanistic target of rapamycin complex 1 (mTORC1) in the phosphatidylinositol 3-kinase (PI3K)/protein kinase B (Akt)/mTOR pathway [45]. The variability of myelin is also dependent on other glial cells, i.e., astrocytes, microglia, and cell types of the vasculature, by providing materials for myelin building and promoting the differentiation of oligodendrocytes [46].

In humans, myelination begins early in the third trimester, but the peak of myelination occurs primarily during the first 2 years after birth. It is mostly complete at 5 years of age but continues until early adulthood in some regions such as the frontal cortex [47]. Myelination follows a fixed spatiotemporal pattern; firstly, myelination appears in the brain stem, followed by the cerebellum and, finally, the cerebral cortex.

A large reservoir of oligodendrocyte progenitor cells persists in the adult brain after the myelination. Oligodendrocyte precursors express a wide array of neurotransmitter and neuroactive ligand receptors. Oligodendrocyte progenitor cells modulate interactions with neurons providing a prominent role in the neuronal activity in the brain [48]. First of all, these cells contribute to physical contact with neurons; secondly, oligodendrocyte progenitor cells are directly connected with glutamatergic and γ-aminobutyric acid (GABA)-ergic neurons that regulate its proliferation. NG2^+^ cells increase the frequency and amplitude of spontaneous glutamatergic inputs during the first 3 postnatal weeks [49], while transmission between GABAergic interneurons and NG2^+^ cells diminished during development [50]. Moreover, α-amino-3-hydroxy-5-methyl-4-isoxazolepropionic acid (AMPA) receptor-dependent signaling in oligodendrocyte lineage cells promotes oligodendrocyte development and myelination during postnatal development [48]. The expression of AMPA receptors in the oligodendrocyte progenitor cells helps to rapidly respond to synaptic input through membrane depolarization and local calcium influx [51]. An increase in the calcium permeability of AMPA receptors via modification of the amino acid composition of the GluA2 subunit enhanced the proliferation of the oligodendrocyte progenitor cells and reduced the number of mature oligodendrocytes [52], while loss of AMPA receptor signaling did not alter the proliferation of the oligodendrocyte progenitor cells but decreased the survival of mature oligodendrocytes [53]. In addition to neurotransmitter receptors to amino acids (glutamate and GABA), oligodendrocyte progenitor cells express receptors for other neuromodulators (ATP, acetylcholine, histamine, norepinephrine, serotonin, dopamine) and neuropeptides (substance P, angiotensin II, bradykinin). Activation of these receptors induces intracellular calcium signaling, suggesting that oligodendroglia can sense and respond to numerous neuronal-activity-dependent signals and that the microenvironment may regulate their cellular behavior [48]. Taken together, myelin and oligodendrocyte lineage cells through different effects on cellular processes may function as a central player to link previously proposed theories of depression and may play a significant role in the pathogenesis of this disease.

## 4. Myelin-Related Changes in Depression

### 4.1. Preclinical Studies

At the preclinical level, impaired myelination is linked to a “depressive-like” phenotype. In fact, in a model induced by chronic unpredictable stress (CUS), a lower volume of white matter [54] and the medial prefrontal cortex [55], along with reduced length and volume of the myelinated fibers and myelin sheath thickness [54], was observed, while a reduced number of oligodendrocytes was seen in the prelimbic cortex [56] in depressed rats. It seems that stress induces myelin structure alterations, which in turn may trigger depression, rather than the depression itself provoking myelin dysfunction. At the same time, the protein expression of CNPase and MBP and the MBP intensity were reduced in the medial prefrontal cortex [55], whereas reduced protein levels of MBP and Olig2 were observed in the hippocampus [57] in these rats (Table 1). Interestingly, a running exercise reversed the depression-like phenotype in stressed animals and had a positive effect on the differentiation of oligodendrocytes, myelinated fibers, and myelin-forming ability in these brain regions [54,55,57].

A similar myelin-related structural effect of chronic stress was seen in the medial prefrontal cortex of mice [60] and rat pups after maternal separation [65]. In fact, hypomyelination and a reduction in the number of oligodendroglial lineage cells and mature oligodendrocytes were presented in this structure, which impaired cognitive functions in mice subjected to chronic stress [60] and in rat pups separated from their dam 3 h daily during the first three postnatal weeks [65]. The lack of maternal care during early life alters myelination in the medial prefrontal cortex immediately after stress exposition and even in adult rats [65]. It should be highlighted that maternal separation reduces histone deacetylases 1/2 (HDAC1/2), and this reduction promotes the Wnt signaling pathway, which in turn negatively regulates the development of oligodendrocytes and impairs the oligodendrocyte precursor cell proliferation and oligodendrocyte maturation [65]. These data highlight the different neurobiological effects of chronic stress exposure on myelin disruption, which may contribute to behavioral changes. Additionally, 4-week chronic variable stress evoked downregulation of genes related to oligodendrocytes and myelin in the medial prefrontal cortex and nucleus accumbens of mice, which was observed even after 1 week of stress with weaker behavioral effects [62]. However, upregulation of myelin-related transcripts was observed in the corpus callosum of mice following 4 weeks of stress [62]. This finding conflicts with data in mice, in which 3 weeks of stress evoked narrowing of the nodes and paranodes of Ranvier, redistribution of contactin-associated protein (Caspr) and voltage-dependent potassium channel (K_v_1.1) in the corpus callosum, and decreased activity in white matter, suggesting the association of morphological changes in oligodendrocytes with an inhibition of axonal activity [61] (Table 1). Additionally, using an oligodendrocyte primary culture subjected to chronic stress, it was shown that altered organization of the nodes of Ranvier was related to inhibition of the transcription of metabotropic glutamate receptors [61].

In a very recent study by Kurokawa and coworkers (2020), major myelin proteins and mature oligodendrocytes were decreased in the hippocampus of stress-maladaptive but not stress-adaptive mice following chronic exposure to restraint stress [70]. On the other hand, stress related to maternal separation 3 h daily during the first three postnatal weeks did not change myelination in the corpus callosum, striatum, or hippocampus at postnatal days (PNDs) 21 and 60 [65], whereas myelination was not altered following 8-week social isolation in the corpus callosum and nucleus accumbens of isolated mice [63]. Prolonged social isolation in mice induced behavioral, transcriptional, and ultrastructural changes in oligodendrocytes in the prefrontal cortex [63,64], in addition to provoking changes within nuclear chromatin condensation as an additional marker related to the maturation of oligodendrocytes, which in turn contributed to impaired adult myelination of the prefrontal cortex [63]. The latter changes were normalized after reintroduction into a social environment only when isolation was in juvenile and adult mice [63]. Contrastingly, mice isolated immediately after weaning evoked myelin alterations in the prefrontal cortex and changes in the prefrontal cortex-dependent cognitive behavior that did not recover upon reintroduction to a regular environment, suggesting that the disturbed myelination during this critical period has functional consequences in adulthood [64] (Table 1).

Reductions in myelin thickness, length of internodes, and myelinated segments in the medial prefrontal cortex were specific to mice that displayed social avoidance behavior (“susceptible” mice) during chronic social defeat stress, while decreased MBP labeling was observed in mice both susceptible and resilient to stress in the nucleus accumbens [69]. Additionally, in the medial prefrontal cortex of susceptible mice, reduced numbers of mature oligodendrocytes were correlated with a decrease in repressive histone methylation marks associated with differentiation (histone 3 lysine 9 trimethylation; H3K9me3) of oligodendrocytes [69]. Another study also confirmed the structural changes in the prefrontal cortex following chronic social defeat stress; the authors highlighted that this kind of stress provokes downregulation of myelin-related genes, and they provided evidence for the molecular basis of altered myelination in this structure [68]. These data parallel observations in the hippocampus and prefrontal cortex in stress-induced (social defeat stress) and genetic (learned helplessness) [67] animal models of depression, where reduced levels of oligodendrocyte precursor cells were observed. It should be noted that social defeat stress reduced NG2 glial secretion of FGF2 in stress-susceptible mice, and the latter change by itself is sufficient to induce depression-like behavior, which was presented using the transgenic line of mice with NG2 ablation, comparable to stress-induced behavior [67]. These data highlight the different myelin-related effects of chronic stress exposure, which could lead to a depression-like phenotype. Moreover, clemastine, an antimuscarinic compound, administered orally for 2 weeks in adult mice following social isolation showed antidepressant-like effects via enhancement of oligodendrocyte progenitor differentiation and epigenetic changes (higher levels of H3K9me3) in the oligodendrocytes of the prefrontal cortex [72]. These data not only link the oligodendrocyte dysfunction with depressive-like behavior but also strongly support the engagement of myelin-related changes in depression and the efficacy of a promyelinating drug in this disorder in preclinical studies.

According to these observations, in which the myelination is damped during depression (above), antidepressant drugs should reverse and/or normalize this alteration. In fact, it was shown that fluoxetine, a selective serotonin reuptake inhibitor, administered chronically reversed the suppressive effect of chronic unpredictable mild stress (CUMS) on myelin-related gene expression in the amygdala [58,59] and cingulate cortex [59]. In contrast, neither chronic defeat stress nor chronic fluoxetine treatment affected the level of newly generated cells differentiated into oligodendrocyte precursor cells (NG2^+^) in the medial prefrontal cortex of rats [66]. Moreover, fluoxetine administered to rhesus monkeys did not change the packing of oligodendrocytes and size of oligodendrocyte cell bodies; however, this drug was administered to naïve animals not introduced into the depression model [73]. Furthermore, imipramine reversed the impaired myelination (reduced levels of myelin-related proteins, number of nodes of Ranvier, and numbers of mature and immature oligodendrocytes) induced by removal of the olfactory bulbs in mice and improved depression-like behavior [71] (Table 1). These data seem to support the participation of the disturbance of myelin function and myelination in the pathophysiology of depression. Because there have been no studies with other antidepressant drugs, further research is urgently needed to validate the exact relationship between antidepressant effects and myelination.

### 4.2. Human Studies

Myelin-related changes in depression were also documented in human studies. In fact, in patients with major depressive disorder (MDD), myelin levels were reduced in the whole brain and nucleus accumbens, whereas, in the lateral prefrontal cortex, myelin levels were decreased in depressed patients with a greater number of depressive episodes [74]. Depression was related to lower white-matter microstructural integrity and gray-matter loss in several brain areas [75,76,77,78]. Additionally, a reduction in the integrity of the macromolecular protein was seen in patients with late-life MDD in white-matter tracts and subcortical nuclei [79], as well as in multiple left-hemisphere frontostriatal and limbic regions, the thalamus, the corpus callosum, and occipital white matter [61,80] compared with controls. Similarly, patients with treatment-resistant depression had a reduced integrity of myelin in task-positive network regions (bilateral precentral gyrus and left middle occipital lobe) and in default-mode network regions (left precuneus and left temporal lobe) [81]. On the other hand, antidepressant treatment evoked greater functional connectivity in the frontal precentral gyrus compared to never depressed old adults, but there was no difference between groups concerning white-matter hyperintensity burden points [82]. A meta-analysis of studies examining white-matter hyperintensities in mood disorder patients showed that depressed patients (i.e., unipolar and bipolar) with a history of suicide had higher deep-white matter hyperintensities and periventricular hyperintensities compared to patients without attempted suicide [83].

Postmortem studies on the brains of depressed patients confirmed the lower intensity of myelin staining in the dorsolateral prefrontal cortex regions in MDD individuals [84] and unipolar and bipolar affective disorders [85]. The reduction in the number of glial cells was significant in subgroups of subjects with MDD or bipolar disorder with a clear family history of depression in the subgenual part of the prefrontal cortex [86]. Additionally, MDD subjects had a greater mean myelin cross-sectional area and myelin thickness per axon in the corpus callosum genu [87], whereas a reduced level of mean myelin cross-sectional area was seen in the splenium of the corpus callosum [88]. A morphometric study also showed a reduction in the numerical density of oligodendroglial cells in the dorsolateral prefrontal cortex [73,89,90,91], frontopolar cortices [92], CA1 pyramidal layer of the hippocampus [93], and amygdala [94] of individuals with MDD, as well as in the prefrontal cortex [89,90,91], caudate nucleus [95], and CA1 pyramidal layer and left alveus of the hippocampus [93] of brains from subjects with bipolar disorder (Table 2). These changes provide evidence for the lowered density of oligodendroglial cells in depression that may contribute to the atrophy of neurons and play a key role in the pathophysiology of this disorder.

The molecular data from human depressed brains show that reduced levels of oligodendroglial cells correlate with downregulation of myelin-related genes and proteins related to transcription factors [67,97,99], oligodendrocyte function [97], myelin synthesis [59,97], and structural components of myelin sheaths [59,73,91,96,97,98,101] (Table 2). In contrast, increased expression of CNPase, OLIG1, and MOG mRNA was observed in the ventral prefrontal cortex of MDD subjects [73]. The authors suggested that these increased levels of gene expression probably compensated for the lower PLP1 level, which is responsible for about 50% of myelin in the CNS [102]. Interestingly, sex-dependent differences and brain-region-specific gene expression for oligodendrocytes were observed in the postmortem depressed brain [100]. Thus, the levels of these genes were either increased in the dorsolateral prefrontal cortex and subgenual anterior cingulate cortex or decreased in the amygdala of men with MDD, whereas, in women with MDD, these effects were opposite (reduced expression in the dorsolateral prefrontal cortex and subgenual anterior cingulate cortex but increased expression in the amygdala) [100]. These data show the opposite molecular signatures of MDD in men and women, which is consistent with the sex-specific changes in incidence, symptomatology, and neuroimaging in MDD patients [103].

Compared to the preclinical study in which genetic ablation of oligodendrocyte precursor cells provoked the depressive-like behavior in mice [67], human studies seem to be less convincing. Thus, studies in humans are correlative but did not demonstrate directly that myelin alterations are causally implicated in the pathogenesis of depression. However, these findings suggest that depressive disorders may be associated with abnormalities in the structural oligodendroglia, while altered gene expression represents a potential molecular mechanism for the degeneration of axons and the dysfunctional maturation of oligodendrocytes in depression.

## 5. Crosstalk among Maternal Malnutrition, Myelination, and Depression

### 5.1. Preclinical Studies

From the preclinical point of view, a modified maternal diet during pregnancy and lactation seems to play a critical role in producing a depression-like phenotype in offspring [6,7,8,9,10]. Since most of the brain development occurs prenatally and during lactation, while oligodendrocytes are extremely sensitive to the alteration in local homeostasis, and since its survival and/or maturation may be limited and arrested as a result of various kinds of pathological signals including environmental effects, the modified diet in mothers may be a crucial factor for programming severe changes in offspring. However, little is known about the effect of diet during gestation and lactation on the myelination in offspring brain.

A maternal high-fat diet 5 weeks before mating and during pregnancy and lactation reduced the level of oligodendrocyte precursors in the lateral cortex of offspring at PND 7 [104]. At the same time, a modified maternal diet evoked decreases in myelination in the medial cortex of male but not female offspring at PND 21 [104]. The latter changes were associated with disruption of iron regulation and inflammatory cytokine homeostasis in the offspring. Interestingly, these structural changes correlated with altered learning and memory behavior [104]. The latter changes were associated with changes in the iron-regulatory proteins (hepcidin, ferroportin, and l-ferritin) and disruption of iron regulation in pups born to high-fat diet-fed dams, which is a crucial component in the progression of myelination in developing neurons. Next, maternal-obesity-induced hepcidin dysregulation was associated with neuroinflammation and oxidative stress in pups at the critical period from birth through 21 days, when active brain growth, extension of neuronal processes, migrating oligodendrocytes, and myelination were the most extensive. Furthermore, an iron-deficient diet during gestation and lactation in female rats evoked a decrease in the diameter of myelinated axons and peak amplitudes of compound action potentials specific to these axons in the corpus callosum of offspring at PND 40, whereas a reduction in the dendritic length of pyramidal neurons in the hippocampus and a decrease in branching complexity in the cortex were observed at PND 21 [105]. Parallel to the structural myelin-related changes, these rats presented deficits in recognition memory at PND 40 [105]. Another study showed that these memory deficits were attenuated by prenatal choline supplementation and were related to the restoration of reduced hippocampal Mbp mRNA levels [106]. These data parallel observations in mouse offspring, which presented loss of social memory and sensorimotor gating deficits at the behavioral level, related to exposure to a high-fat diet in mothers for 4 weeks before mating, during gestation, and until weaning [107]. Reductions in the number and area of myelin cytosolic channels in the rostrum of the corpus callosum of adolescent male mouse offspring were found, whereas, in the hippocampus, a key projection region of the corpus callosum, the myelination-associated transcripts and myelin-promoting growth factors were decreased [107]. The above sex-specific transcriptomic changes are in line with the evidence suggesting that maternal-diet-induced epigenetic regulation in the brain is often sex-specific and depends on estrogen receptor expression, neuroinflammatory signals, neonatal hormone exposure, and cellular differences in genetic sex [108]. Additionally, reduced numbers of mature lysosomes and increased synaptic contacts were observed in the corpus callosum of offspring following a maternal high-fat diet without effects on the processes involved in the density, distribution, or maturation of oligodendrocytes, which suggests that maternal-diet-induced changes within the microglia may be involved in the modified myelination [107]. On the other hand, alcohol exposure during gestation and lactation in dams induced an alteration of myelin damage biomarkers in the prefrontal cortex, and this effect was mitigated by high-fat diet feeding during childhood and adolescence in female offspring, suggesting that the fatty diet may supply extra lipids that could overcome the deleterious effects of alcohol on myelination [109]. On the other hand, a diet enriched in n-3 polyunsaturated fatty acids (ꞷ-3 acids) in mice 2 months before conception and continued throughout lactation dose-dependently increased the expression levels of MBP in pup mice at PND 21 and 42 [110] and decreased apoptosis and hypomyelination in the lipopolysaccharide-induced white-matter injury [111], suggesting that maternal ꞷ-3 acids promote early brain development. Higher levels of docosahexaenoic acid (DHA) in the maternal diet increased the myelin content, altered the lipid composition of rat pup myelin, and increased latencies of the auditory startle response in rat pups [112]. Cotreatment with essential fatty acids and zinc during pregnancy led to greater MBP levels in the brainstem of piglets compared to the control or each supplement alone [113]. Similarly, lactoferrin, a component of maternal milk, supplemented in maternal food during lactation displayed neuroprotective effects on reduced lipopolysaccharide-induced ventriculomegaly, brain tissue loss, and microstructural modifications, including myelination deficit in mice pups [114].

In the latest study by Trujillo-Villarreal and coworkers (2021), it was shown that a cafeteria diet in mothers for 9 weeks (pre-pregnancy, pregnancy, and lactation) might prime depression-like behavior in the offspring, observed as a deficient motivation for natural rewards [115]. Structural studies presented a reduction in the local volume of the hippocampus, nucleus accumbens, and thalamus in offspring [115]. At the same time, a reduction in synaptic terminals, cell number, and myelin staining was seen in the hippocampus, which was accompanied by dysregulation in the hippocampal glutamatergic system [115]. These data seem to support the participation of maternal malnutrition in the myelin-related changes and depression-like phenotype in offspring.

Interestingly, chronic nutritional stress using a low-calorie protein diet in mothers during pregnancy and lactation produced long-term changes in network parameters in offspring brain [116] and in the optic nerve [117]; however, this altered diet did not change the long integrative myelinated tracks in mice pups, but it reduced the frequency of short tracks in the central brain regions [116]. The rats fed an iodine-deficient diet from 3 months before pregnancy to the end of lactation altered the hippocampal myelin at PND 14 and 21, whereas hypothyroxinemia reduced the expression of Olig2 and myelin-related proteins in these rats, suggesting that impairment of hippocampal myelinated growth may cause the neurological deficits and alterations of brain function in offspring [118].

In summary, since most brain development occurs prenatally and during lactation, maternal nutrition has been identified as a key factor for brain growth and maturation in offspring. According to preclinical research, a modified maternal diet provokes different myelin-related changes in rodents including gene and/or protein expression related to myelin, maturation of oligodendrocytes, and myelin structure changes, as a cellular mechanism that may contribute to behavioral alterations in offspring. Moreover, further research is needed to elucidate the molecular mechanisms related to myelin responsible for the development of depressive-like behavior in offspring exposed to a modified maternal diet, as well as search for contributory factors in the development of mental brain disorders.

### 5.2. Human Studies

In humans, there are several lines of evidence that maternal malnutrition is implicated in myelin-related alterations. A case study on a 9-month-old girl with vitamin B12 deficiency, whose mother was a strict vegetarian with a low socioeconomic status, showed psychomotor regression, hypotonia, and lethargy with cerebral atrophy and delayed myelination [119]. A similar study on a 5-month-old Italian male infant hospitalized because of poor weight gain, feeding difficulties, severe pallor, muscle hypotonia, and somnolence led to a diagnosis of having vitamin B12 and iron deficiency due to nutritional inadequacy (mother was a vegan treated with a multivitamin oral preparation during the second and third trimesters). This maternal malnutrition provoked mild dilatation of the lateral ventricles with diffuse delayed myelination in the brain [120], suggesting that altered myelin formation and integrity are related to dysregulated incorporation of fatty acids, as well as an accumulation of lactate or neurotoxic cytokines. These data only link maternal diet with myelination; however, further research is urgently needed to validate the clinical relationship between maternal diet and risk of depression in children.

The behavioral changes observed in early childhood may be associated with morphological, molecular, and functional alterations in the brain after a modified maternal diet. Importantly, several mechanisms including epigenetic (i.e., posttranslational modifications of histone proteins, noncoding RNAs, and DNA methylation), inflammatory, and hormonal may contribute to individual differences in predisposition to depression; however, further investigations are needed. In this regard, the maternal diet during gestation and lactation can lead to long-lasting alterations in the epigenome, which may induce long-term neurobiological modifications affecting synaptic function and structural plasticity.

## 6. Conclusions

This review can raise awareness regarding the higher risk of developing problems during delivery of children (including the transmission of disorders), thus allowing for a better understanding of the mechanisms of such transmission and ultimately preventing it. In fact, the diet is one of the main sources of developmental brain damage with possible long-life neurodevelopmental disabilities; thus, understanding the effects of maternal diet on the developing brain as well as on depression is an important public health concern worldwide. Poor antenatal and postnatal nutrition further prevents infants from attaining their full developmental potential and developing affective cognition and increases their susceptibility to psychiatric disorders. As presented in this review, preclinical and clinical studies show that depression is associated with myelin-related changes. Additionally, maternal malnutrition during pregnancy and lactation is associated with specific alterations in the myelination processes in offspring, which seem to contribute to neurobehavioral changes in adolescence and adulthood, including a depression-like phenotype. However, more studies are required to determine the exact outcome of this problem to break new ground in our understanding of the impact of a maternal high-fat diet during gestation and lactation on myelination in brain offspring and to provide novel mechanistic strategies underlying myelin modulation in the pathogenesis of depression.

## Figures and Tables

**Table 1:** Myelin-related changes in depression: animal studies.

Animal Model of Depression	Animal Sex	Molecular Effect/Antidepressant Effect (Dose, Treatment, Route)	References
CUS	Sprague–Dawley rats; males	- ↓ number of oligodendrocytes—prelimbic cortex	[56]
	Sprague–Dawley rats; males	white matter: - ↓ total volume - ↓ total length and volume of the myelinated fibers - ↓ total volume of the myelin sheaths - ↓ myelin sheath thickness - ↓ outer diameter of the myelinated fibers	[54]
	Sprague–Dawley rats; males	HIP: - ↓ MBP, Olig2 (protein) - ↓ CNPase^+^ cells number—CA3, dentate gyrus	[57]
	Sprague–Dawley rats; males	mPFC: - ↓ volume - ↓ MBP, CNPase (protein) - ↓ MBP intensity - ↓ CNPase^+^ cells number	[55]
CUMS	BALB/c mice; males	amygdala: - ↓ oligodendrocyte transcripts	[58]
		fluoxetine (20 mg/kg; 36 days; i.p.): reversed	
	BALB/c mice; males	altered gene expression - 254 genes—cingulate cortex - 299 genes—amygdala	[59]
		fluoxetine (20 mg/kg; 36 days; i.p.): - 75% genes reversal—cingulate cortex - 72% genes reversal—amygdala	
chronic stress	C57BL/6N mice; males	mPFC: - atrophy NG2^+^ cells—↓ segments, ↓ branching, ↓ terminal points, shorter dendrite, ↓ volume - ↓ number of oligodendroglial lineage cells (Olig2^+^) - ↓ newly generated ligodendroglial lineage cells (Olig2^+^ BrdU^+^) - ↓ number of mature oligodendrocytes - ↑ Casp3 in oligodendrocytes - ↓ MBP labeling (hypomyelination)	[60]
	C57/BL6 mice; males	corpus callosum: - ↓ lengths of axon nodes and paranodes - ↓ Caspr immunoreactivity in the paranodes of Ranvier - ↑ Caspr length - ↓ node width (length of Caspr-positive structures) - ↓ areas of K_v_1.1 immunoreactivity in juxtaparanodes of Ranvier - ↑ K_v_1.1 length - ↑ Caspr, contactin, TAG1 (protein) - ↓ neurofascin (protein) - ↓ ATPase activity, ATPase Na^+^/K^+^ transporting	[61]
	C57B1/6 mice; males	4 weeks of stress: - ↓ MAG, MBP, MOBP, Tfr, Cspg4, Pdgfra, Olig2 (mRNA)—mPFC - ↓ MAG, MOBP, Cspg4, SOX10, Olig2 (mRNA)—NAc - ↑ MOG, MBP, Cspg4 (mRNA)—corpus callosum 3 weeks of stress: - Ø—mPFC - Ø—NAc - ↓ MAG, MBP, MOBP, PLP (mRNA)—corpus callosum 2 weeks of stress: - ↓ Olig1, Olig2 (mRNA)—mPFC - ↓ MAG, MOBP (mRNA)—NAc - Ø—corpus callosum 1 week of stress: - ↓ MAG, Cspg4, Olig1, Olig2 (mRNA)—mPFC - ↓ MAG, MOBP, Tfr, Qki, Cspg4, Pdgfra (mRNA)—NAc - ↓ Olig1—corpus callosum	[62]
social isolation	C57B1/6J mice; males	PFC: 8 weeks of isolation: - ↓ oligodendrocyte-specific paranodal genes (NFasc155, Cntn2) - ↓ myelin thickness - ↓ MBP (mRNA, protein), MOBP (mRNA), MOG (mRNA) 15 days of isolation: - ↓ myelin thickness	[63]
	crossing male FVB/N mice and female C57Bl/6 mice; males	mPFC: isolation from PND 21 to PND 65: - Ø oligodendrocyte density - oligodendrocytes with simpler morphology - ↓ MBP, MAG (mRNA) - ↓ myelin thickness isolation from PND 21 to PND 35, then returned to regular environment until PND 65: - Ø oligodendrocyte density - oligodendrocytes with simpler morphology - ↓ MBP, MAG (mRNA) regular environment from PND 21 to PND 35, then isolation until PND 65: - Ø	[64]
maternal separation	Sprague–Dawley rats; females and males	mPFC: - ↓ MBP (PND 7, 21, 60) - ↓ myelin sheath thickness (PND 21, 60) - ↑ number of oligodendrocyte precursor cells (NG2^+^) (PND 21) - ↓ number of mature oligodendrocytes (Olig2^+^ CC1^+^) (PND 21) - ↓ oligodendroglial lineage cells (Olig2^+^) proliferation (PND 7, 10, 14, 21) - ↑ number of newly generated oligodendrocyte precursor cells (NG2^+^ BrdU^+^) (PND 21) - ↓ number of newly generated oligodendrocytes (CC1^+^ BrdU^+^) (PND 21) No changes (PND 21, 60)—corpus callosum, striatum, HIP	[65]
chronic social defeat stress	Wistar rats; males	mPFC: - Ø number of NG2^+^ cells	[66]
		fluoxetine (10 mg/kg; 28 days; p.o.): Ø	
	C57BL/6J mice; males	PFC: - ↑ NG2 glia density (PDGFRα^+^ cell number) (4 days of defeat) - ↓ NG2 glia density (PDGFRα^+^ cell number) (8 days of defeat; 8 days of defeat +10 days post-defeat) CA1 of HIP: - ↓ NG2 glia density (PDGFRα^+^ cell number) (4 days of defeat; 8 days of defeat; 8 days of defeat + 10 days post-defeat)	[67]
	C57BL/6 mice; males	mPFC: - ↓ 74 myelin-related genes (transcript) - ↓ MOG, MOGP, Trf, Ermn (mRNA) - ↓ MOG mRNA^+^ cells - ↓ Ermn mRNA^+^ cells - ↓ myelinated fiber length and density - ↓ MBP staining	[68]
	C57B1/6J mice; males	susceptible mice mPFC: - ↓ length of myelinated segments - ↓ internodal length - ↓ myelin thickness - ↑ number of NG2^+^ progenitor cells - ↓ number of CC1^+^ mature oligodendrocytes susceptible and resilient mice NAc: - ↓ MBP labeling	[69]
chronic restraint stress	ICR mice; males	HIP: stress-maladaptive mice: - ↓ MAG, MBP (protein) - ↓ density of CC1^+^ mature oligodendrocyte cells—dentate gyrus - ↓ number of CC1^+^/Olig2^+^ cells—dentate gyrus	[70]
learned helplessness	Sprague–Dawley rats; males	↓ NG2 glia density (PDGFRα+ cell number)—PFC	[67]
olfactory bulbectomy	ddY mice; males	14 days after surgery: PFC: - ↓ MBP, MAG (protein) 21 days after surgery: PFC: - ↓ MBP, MAG, CNPase (protein) - ↓ nodes of Ranvier - ↓ Caspr level - ↑ NG2^+^/Olig2^+^ cells - ↓ CC1^+^/Olig2^+^ cells striatum: - ↓ MBP (protein)	[71]
		imipramine (20 mg/kg; 14 days; i.p.): PFC: reversed ↓ MBP, ↓ MAG, ↓ CNPase, ↓ Caspr, ↓ number of nodes, ↓ NG2^+^/Olig2^+^, ↓ CC1^+^/Olig2^+^, ↓ Olig2^+^ cells	

↑, increased; Ø, no change; ↓, decreased; +, positive; Casp3, caspase 3; Caspr, contactin-associated protein; CC1, adenomatous polyposis coli (APC) clone CC1; CNPase, 2′,3′-cyclic nucleotide 3′ phosphodiesterase; Cspg4, chondroitin sulfate proteoglycan 4; CUMS, chronic unpredictable mild stress; CUS, chronic unpredictable stress; Ermn, ermin; HIP, hippocampus; i.p., intraperitoneal; K_v_1.1, voltage-dependent potassium channel; MAG, myelin-associated glycoprotein; MAL, mal, T-cell differentiation protein; MBP, myelin basic protein; MOBP, myelin-associated oligodendrocyte basic protein; MOG, myelin oligodendrocyte glycoprotein; mPFC, medial prefrontal cortex; NAc, nucleus accumbens; NG2, neuron–glial antigen 2; Olig1, oligodendrocyte transcription factor 1; Olig2, oligodendrocyte transcription factor 2; PDGFRα (a), platelet-derived growth factor receptor α (a); PFC, prefrontal cortex; PND, postnatal day; p.o., per os; QKI, quaking; SOX10, SRY-related HMG-box 10; TAG1, transient axonal glycoprotein 1; Trf, transferrin.

**Table 2:** Myelin-related changes in depression: human postmortem studies.

Disease	Study Sample Size	Molecular Effect	References
MDD, BPD	9 MDD; 14 BPD; 11 C	↓ glial number—familial subgroup—subgenual PFC	[86]
depression with suicide	11 (10 C)	↓ MBPD (protein)—anterior PFC	[96]
MDD, BPD	15 MDD; 15 BPD; 15 C	↓ density of oligodendroglial cells—layer VI dorsolateral PFC	[89]
MDD, BPD	8 MDD; 9 BPD; 10 C	↓ density of glia and oligodendrocytes—MDD—amygdala	[94]
MDD	12 MDD (14 C)	↓ expression of genes for: - transcription factors (OLIG2, SOX10) - oligodendrocyte differentiation (ERBB3) - myelin synthesis (ASPA, UGT8, ENPP2, EDG2, KLK6), - structural myelin components (CNPase, MAG, MAL, MOG, MOBPD, PMP22, PLLP, PLP1)—temporal cortex	[97]
MDD, BPD	11 MDD; 10 BPD; 14 C	↓ NOGO-B (mRNA)—frontal cortex	[98]
MDD, BPD	15 MDD; 15 BPD; 15 C	↓ number of perineuronal oligodendrocytes—sublayers IIIa, IIIb, IIIc dorsolateral PFC	[90]
BPD, unipolar MD	15 MDD; 15 BPD; 15 C	↓ mean deep white matter myelin staining—dorsolateral PFC	[85]
MDD	16 MDD; 13 C	↓ QKI (mRNA)—cortex, hippocampus, amygdala	[99]
MDD	14 MDD; 14 C	↓ CNPase (mRNA, protein) ↓ MBPD, PLLP, MOBPD, GPR37, ENPP2 (mRNA)—amygdala	[59]
MDD, BPD	12 MDD; 13 BPD; 15 C	↓ MOG, OMG, PLP1 (mRNA) ↓ number of perineuronal oligodendrocytes—dorsolateral PFC BA9	[91]
MDD	11 MDD; 12 C	↓ oligodendroglial cells—frontopolar cortices BA10	[92]
MDD, BPD	9 MDD; 6 BPD; 13 C	MDD, BPD: ↓ density of S100B-immunopositive astrocytes—CA1 piramidal layer BPD: ↓ density of S100B-immunopositive oligodendrocytes—left alveus	[93]
MDD	16 MDD; 20 C	↑ mean myelin cross-sectional area ↑ myelin thickness—corpus callosum genu	[87]
MDD	12 MDD; 8 C	↓ cell numbers of NG2 glia ↓ PDGFRα (protein)—frontal cortex	[67]
MDD	20 MDD; 16 C	↓ oligodendrocyte soma size ↓ size of oligodendrocyte cell bodies—ventral PFC gyral white matter ↓ PLP1 (mRNA) ↑ CNPase, MOG, Olig1 (mRNA) ↓ CNPase (protein)—ventral PFC white matter	[73]
MDD, BPD	15 MDD; 15 BPD; 15 C	MDD: ↓ intracortical myelin staining—dorsolateral PFC	[84]
MDD	26 MDD men; 24 MDD women; 50 C	men: ↑ oligodendrocyte-related genes women: ↓ oligodendrocyte-related genes—dorsolateral PFC, anterior cingulate cortex, basolateral amygdala	[100]
depression	18 depression; 18 C	↓ MBPD (protein)—ventromedial PFC white matter	[101]
MDD	16 MDD; 20 C	↓ mean myelin cross-sectional area—corpus callosum splenium	[88]
MDD, BPD	15 MDD; 15 BPD; 15 C	BPD: ↓ numerical density of oligodendrocytes and oligodendrocyte clusters—caudate nucleus	[95]

↑ increased; ↓ decreased; BA, Brodmann area; BPD, bipolar disorder; C, control; CNPase, 2′,3′-cyclic nucleotide 3′ phosphodiesterase; EDG2, endothelial differentiation, lysophosphatidic acid G-protein-coupled receptor, 2; ENPP2, ectonucleotide pyrophosphatase/phosphodiesterase 2; ERBB3, erb-b2 receptor tyrosine kinase 3; GPR37, G-protein-coupled receptor 37; KLK6, Kallikrein-related peptidase 6; MAG, myelin-associated glycoprotein; MAL, mal, T-cell differentiation protein; MBP, myelin basic protein; MDD, major depressive disorder; MOBP, myelin-associated oligodendrocyte basic protein; MOG, myelin oligodendrocyte glycoprotein; NG2, neuron–glial antigen 2; NOGO, reticulon 4; OLIG1, oligodendrocyte transcription factor 1; OLIG2, oligodendrocyte transcription factor 2; OMG, oligodendrocyte myelin glycoprotein; PFC, prefrontal cortex; PLLP, plasmolipin; PLP1, proteolipid protein 1; PMP22, peripheral myelin protein 22; QKI, quaking; UGT8, UDP glycosyltransferase 8.

## Data Availability

Not applicable.
